# Credentialing for participation in clinical trials

**DOI:** 10.3389/fonc.2012.00198

**Published:** 2012-12-26

**Authors:** David S. Followill, Marcia Urie, James M. Galvin, Kenneth Ulin, Ying Xiao, Thomas J. FitzGerald

**Affiliations:** ^1^Radiological Physics Center, Department of Radiation Physics, University of Texas MD Anderson Cancer CenterHouston, TX, USA; ^2^Quality Assurance Review Center, Department of Radiation Oncology, University of Massachusetts Medical SchoolLincoln, RI, USA; ^3^Department of Radiation Oncology, Jefferson Medical College, Thomas Jefferson UniversityPhiladelphia, PA, USA; ^4^Radiation Therapy Oncology GroupPhiladelphia, PA, USA; ^5^Department of Radiation Oncology, University of Massachusetts Medical SchoolWorcester, MA, USA

**Keywords:** credentialing, clinical trials, radiation oncology, quality assurance, clinical cooperative groups

## Abstract

The National Cancer Institute (NCI) clinical cooperative groups have been instrumental over the past 50 years in developing clinical trials and evidence-based clinical trial processes for improvements in patient care. The cooperative groups are undergoing a transformation process to launch, conduct, and publish clinical trials more rapidly. Institutional participation in clinical trials can be made more efficient and include the expansion of relationships with international partners. This paper reviews the current processes that are in use in radiation therapy trials and the importance of maintaining effective credentialing strategies to assure the quality of the outcomes of clinical trials. The paper offers strategies to streamline and harmonize credentialing tools and processes moving forward as the NCI undergoes transformative change in the conduct of clinical trials.

## INTRODUCTION

The national cooperative group clinical trials system is more than 50 years old. In the beginning the newly established cooperative groups placed emphasis on clinical trials involving leukemia and lymphoma. As these trials matured and acquired the confidence and enthusiasm of the clinical oncology community, clinical trials in epithelial oncology began to mature. Accordingly, radiation therapy became incorporated as an important discipline in the clinical cooperative group community. In 1968, the Committee for Radiation Studies recognized the need for consistency in the delivered radiation dose to the patient. The committee was made aware of significant variations in computation algorithms used to calculate radiation dose as well as significant variability in the delivery of daily radiation treatment. In 1969, the Radiologic Physics Center (RPC) was funded by the National Cancer Institute (NCI) with the mission to assure both the NCI and clinical cooperative system that institutions participating in the clinical trials process deliver comparable and consistent radiation therapy compliant to study objectives. Herring and Compton (1970) published an analysis on patients treated for laryngeal carcinoma in 1970 indicating that radiation therapy had to be delivered to a specific radiation dose and volume within 5% of the intent of treatment in order to achieve optimal clinical outcome, a paradigm that remains as important today as it was in 1970. In early clinical trials there was considerable variability identified in both radiation dose and volume of treatment with many major deviations identified on multiple studies (**Table 1**) making interpretation of the study difficult to assess. To further complicate matters, there was no clear mechanism in place to acquire and review protocol data in a central location, making analysis limited and incomplete. It was challenging to use these studies as a vehicle to identify a standard of care. The Quality Assurance Review Center (QARC) was established in 1976 as a subset of the radiation therapy committee of the Cancer and Leukemia Group B (CALGB) with a responsibility to acquire radiation therapy protocol information, review data with responsible clinical trial investigators, and provide institutional feedback on protocol performance. The office offered similar service for the National Wilms Tumor Study Group (NWTSG) and the Intergroup Rhabdomyosarcoma Study Group (IRSG). QARC became independently funded through Cancer Therapy Evaluation Program (CTEP) in 1980 when the pediatric divisions of both CALGB and Southwest Oncology Group (SWOG) separated from the adult Cooperative Groups and formed the Pediatric Oncology Group (POG). QARC and RPC have been continuously funded to date with quality assurance service portfolio evolving as clinical protocols have matured (FitzGerald et al., 2008; Ibbott et al., 2008). The Radiation Therapy Oncology Group (RTOG) developed an internal mechanism for quality assurance as part of the primary program. The image-guided therapy center (ITC) was developed to work in collaboration with the RTOG initially as the resource for three-dimensional planning and has worked in close collaboration with the RTOG since that time point (Eisbruch et al., 2010). All four organizations are housed under the general umbrella of the Advanced Technology Consortium (ATC) designed to establish transparent guidelines and standards in radiation oncology throughout all of the cooperative groups. By 2014, all radiation therapy and diagnostic imaging quality assurance centers will be integrated as a single entity called the Imaging and Radiation Oncology Core (IROC) as part of the re-organization of the clinical trial effort managed by the NCI.

**Table 1:** Comparison of deviation rates for clinical trial studies with and without radiotherapy credentialing.

Clinical trial group	Disease site	Credentialing	Major deviations	Minor deviations	Number of patients
GOG	Endometrial	No	29 (15%)	37 (19%)	197
NSABP	Breast	No	135 (9%)	214 (15%)	1460
RTOG	Cervical	No	43 (14%)	76 (24%)	315
RTOG	H&N	No	75 (7%)	188 (18%)	1073
GOG	Cervical (LDR)	No	57 (21%)	87 (32%)	275
GOG	Cervical (HDR)	Yes	0	15 (21%)	70
COMS	Ocular	Yes	43 (5%)	47 (4%)	1166
RTOG	Breast	Yes	0	4 (4%)	100
RTOG	Prostate	Yes	0	6 (5%)	117
NSABP	Breast	Yes	3 (0.3%)	172 (11%)	1612

Review of data from studies for protocol quality assurance management initially was solely performed in a retrospective manner well past completion of the study. As a result, deviation rates on study were (and often remain) unacceptably high and only captured in retrospect. The approach and concept development for quality assurance had to be re-engineered to address the issues surrounding both computational and volumetric deviations on study (FitzGerald, 2012). In 1985, the Collaborative Ocular Melanoma Study (COMS) recognized the need for a more rigorous quality assurance process to reduce the deviation rate for this disease. Ophthalmologists, radiation oncologists, medical physicists, and statisticians developed a process to vet both individuals and institutions and credential them prior to entering a patient on study. The purpose of this process was to insure to clinical trial investigators and sponsors that the medical team and the institution had the necessary equipment, resources, radiation dose computational tools and algorithms, treatment expertise, and understanding of both the requirements and expectations of the protocol. This required completion of a facility questionnaire, completion of a knowledge assessment test case, submission of previous cases treated in a protocol format, and the viewing of a treatment training video. Due in large part to the credentialing process coupled with continuous feedback to investigators, the deviation rate on these studies was less than 5%. This was a very important contribution as the process created a uniform study population serving to strengthen the validation of the results of the clinical studies (Pettersen et al., 2008). Data on a series of protocols in Hodgkins lymphoma reviewed at QARC demonstrate the importance of real-time intervention of both imaging and radiation therapy treatment objects for compliance to study radiation dose/volume trial objectives (FitzGerald et al., 2008; FitzGerald, 2012).

In the past decade, cooperative group protocols involving the treatment of cervical cancer permitted institutions to treat patients with both low dose rate brachytherapy (LDR) and high dose rate brachytherapy (HDR). Because LDR was considered standard therapy and well established, decision was made not to perform quality assurance (QA) on patients treated with LDR. It was presumed that LDR therapy had been performed for many years and an assumption was made that treatment standards in the community were uniform. HDR therapy was new and less familiar to the therapy population and decision was made to establish a strong credentialing program including HDR source calibration and submission of two patient cases treated in a similar manner to the protocol submitted for dosimetry and clinical review including validation of the planning system calculation parameters for the intended radiation dose delivery. There was no review of LDR brachytherapy treatments prior to or during the time that patients were entered on study. At closure of the trial, there were no major deviations in the HDR cohort that had undergone the rigorous credentialing process. However, as part of retrospective review of treatment objects, there were 57 (21%) patients with major deviations treated with LDR brachytherapy. This influenced trial analysis. **Table 1** lists several NCI clinical trials that had a credentialing requirement or not listed with percent major and minor deviation. As can be seen in studies with credentialing portfolio the deviation rate was considerably decreased in many diverse disease-based studies with a strong credentialing program in place.

The benefit of credentialing is also illustrated by the deviation rates in two head and neck studies that were conducted under industry sponsorship and for which QARC coordinated the quality assurance reviews. Both studies were conducted under the same sponsor, and both included significant international participation. Institutions were credentialed for the first study by completing a “generic” benchmark, which tested some basic treatment planning skills but was not specific to the study in question and did not test the investigator’s expertise in drawing treatment objects for head and neck tumors. The major deviation rate for that study was 24%. Because of this high deviation rate, it was decided to use a protocol-specific benchmark to credential institutions for a follow-up study. For the follow-up study participants were provided with an anonymized CT scan of a select patient that was typical of the patients expected to be seen on the study. They were asked to develop a treatment plan following protocol guidelines, draw all target volumes of interest, and submit all of the data that would be required for an actual protocol patient. The benchmarks were then reviewed exactly as a plan for a protocol patient would be reviewed, including review of target volume delineation as well as the treatment plan itself. The major deviation rate for the follow-up study was only 8%, significantly less than for the initial study.

The concept of what constitutes credentialing has evolved since first introduced and as new radiotherapy delivery technologies, including intensity modulated radiation therapy (IMRT), stereotactic body radiation therapy (SBRT), volume modulated arc therapy (VMAT), and proton therapy, have been included in clinical trials (Summers et al., 2011). Credentialing should not be confused with ongoing periodic QA activities administered by the various QA centers. Examples of these ongoing QA activities include protocol development, monitoring of the participating institution’s therapy beam outputs (optically-stimulated luminescent/thermoluminescent dosimeter, OSLD/TLD program), completion of generic benchmark cases, on site audits at participating institutions and retrospective clinical and dosimetric review of protocol patient treatments. Credentialing is the examination and review of the institution and/or its staff as to whether they meet certain criteria for participation in a specific clinical trial (FitzGerald et al., 2010). Failure to meet the criteria may limit the participation of an institution in a specific protocol. However, the mission of the QA centers is not to restrict participation but to assist institutions in any required remedial actions so that they meet the criteria and can treat radiotherapy patients in a consistent manner consistent with other participating institutions. Credentialing requirements may include the following but, are not limited to since they evolve with each new protocol.

1. Completion or update of a facility questionnaire
2. Demonstration of the ability to transfer radiotherapy data electronically from the institution to a central quality assurance office
3. Completion of a protocol knowledge assessment
4. Submission and review of previously treated patients treated in a manner required by the protocol
5. Completion of a treatment planning protocol-specific benchmark case(s)
6. Completion of a modality and/or site-specific benchmark case
7. Completion of an end-to-end phantom irradiation study
8. Completion of an Image Guidance study
9. Pretreatment clinical and/or dosimetric review of an individual protocol patient (rapid review)

The goal of credentialing is fourfold: (1) educate the institution and its staff as to the requirements for treating the specific protocol patient, (2) verify that the institution can indeed perform the required radiotherapy treatment accurately, (3) provide feedback to the institution on how to correct errors or improve their radiotherapy treatment technique, and (4) minimize the number of patients scored as deviations. As part of the process of clinical trial development, decisions are made concerning the appropriateness of both credentialing and data review. There are clinical trials where data capture is the only aspect of chart review and certain protocols (CNS leukemia, etc) do not have an assigned credentialing or radiation therapy (RT) object review objective. This provides the appropriate distribution of resources for the clinical trial effort.

In 1997, the ATC was funded by the NCI to create a forum through which the QA centers, QARC, RPC, ITC, and RTOG QA, can work together to improve the quality of the clinical trial radiotherapy data for all study groups. The ATC has played a key role in unifying the credentialing activities and creating reciprocity of credentialing among all study groups. Through ATC efforts credentialing guidelines have been introduced for new technologies such as recently for proton therapy (http://rpc.mdanderson.org/RPC/home.htm). The reasoning for each of the possible current credentialing requirements is as follows.

## FACILITY QUESTIONNAIRE

As target volume definition in protocols has become image-driven, accelerator technology has become more complex and radiation delivery modalities change, demonstration of the expertise of use, appropriate comprehensive QA program, equipment/resources necessary to deliver the radiotherapy treatment accurately is required (Cui et al., 2012). To that end, institutions are asked to complete or modify an existing facility questionnaire (FQ) to document the institution’s current state of practice. In the past there have been multiple FQ originating from the study groups and quality assurance offices. Currently, there is an effort to implement a single electronic FQ appropriate for all study groups and quality assurance offices that is web-based. The electronic FQs sent to the institution already completed with information from previous FQ submissions. The institution only needs to edit their FQ to add new information or to delete outdated information. The study groups and quality assurance offices will also have access to this confidential information.

## ELECTRONIC TRANSFER OF RADIOTHERAPY DATA

A critical component of providing QA services for radiation therapy is the transfer of required data to the QA centers. Since the mid-1990s, the ITC located in St. Louis has worked with treatment planning vendors to encourage them to implement export of volumetric dose and structures, together with the planning CT scan. Originally the format of the digital data export was RTOG data exchange format. This is now been largely supplanted by Digital Imaging and Communications in Medicine (DICOM) RT. In large measure as a result of the efforts of the ITC, nearly all commercial planning systems as seen in **Table 2**, can now export volumetric digital data in one of these two transfer formats. The QA centers have developed the ability to import and view this data which enables comprehensive reviews to be performed by QA center staff and/or protocol investigators. Because of the electronic nature of these data submissions, the QA reviews can be performed remotely by investigators as well as at a QA center. Currently, most NCI protocols require electronic data submission and institutions are required to demonstrate the ability to transfer data electronically before being allowed to enter patients onto the trial. Submission and retention of the electronic data not only aids in the review of the radiotherapy data for QA purposes, but also archives the information for secondary analyses to be performed after the trial is complete.

**Table 2:** Treatment planning systems with the capability to transfer data electronically to the ITC.

Treatment planning systems			Exchange format	Treatment modality				
Vendor	System	Version^1^		3DCRT	IMRT	Seed brachy	HDR brachy	Protons
Accuray	MultiPlan	1.5.2	D		Yes			
BrainLAB	iPlan	4.1^2^	D	Yes	Yes			
Elekta	CMS XiO	3.1	R	Yes	Yes	Yes		Yes
	CMS XiO	4.3.1	D	Yes	Yes			
	RenderPlan 3D		R	Yes				
	PrecisePlan	2.01	D	Yes	Yes			
Nomos	Corvus		R		Yes^3^			
Nucletron	Helax TMS		R	Yes	Yes			
	TheraPlan Plus		R	Yes				
	Oncentra MasterPlan	1.5	D	Yes	Yes			
		3.1	D	Yes	Yes		Yes	
	PLATO RTS	2.62	D	Yes				
	PLATO BPS	14.2.6	D				Yes	
	SPOT-PRO	3.1-00	D			Yes		
PerMedics	Odyssey	4.8.02	D	Yes	Yes			Yes
Philips	Pinnacle^3^		R	Yes	Yes			
	Pinnacle^3^	8.0h	D	Yes	Yes			
	AcqPlan	4.9	R	Yes				
Prowess	Panther	4.41	D	Yes	Yes	Yes		
Rosses Med.	Strata Suite CTPlan	4.0	R			Yes		
RTek	PIPER	2.1.2	R			Yes		
TomoTherapy	Hi-ART	3.0	D		Yes			
Varian	BrachyVision	6.5	D				Yes	
	Eclipse	7.1	D	Yes	Yes			Yes
	VariSeed	7.1	D			Yes		

*Exchange formats: D, DICOM RT objects; R, RTOG data exchange format.*

1 Earliest compliant version of treatment planning system.

2 Please contact BrainLAB for data export procedure.

3 Requires use of work-around for ATC-compliant submission.

## KNOWLEDGE ASSESSMENT

The Knowledge Assessment (KA) is a QA tool that requires the radiation oncologist and/or medical physicist to have read the protocol to answer the specific KA questions. These questions cover topics such as dose prescription, treatment techniques, data required to be submitted for each patient, allowed modalities, etc. The KA, as a part of credentialing, improves the quality of the trial by means of education and makes more certain that there is a common lexicon for protocol associated language. Many institutions believe that their local radiotherapy treatment techniques are sufficient to meet the protocol specifications and as such do not fully appreciate all of the protocol specifications. By completing the KA, study groups and QA centers have the confidence that each institution and/or their clinicians, dosimetrists, and medical physicists understand what is specified by the protocol to enter an appropriately treated patient onto the trial instead of a patient destined to result in a deviation. There are several tools in development to help investigators draw treatment objects in various disease sites. These tools will be used as benchmark tests.

## SUBMISSION AND REVIEW OF A PREVIOUSLY TREATED PATIENT, TREATED IN A MANNER REQUIRED BY A PROTOCOL

The submission of previously treated patients in the manner specified by a particular protocol enables the institution to demonstrate to the protocol Principal Investigators (PIs) and QA centers that they have the ability to follow the protocol correctly. They have simulated an actual protocol patient and received feedback on how to make any needed treatment adjustments before even attempting to treat the protocol patient. The key component to this credentialing requirement is the feedback given to the institution. This particular credentialing requirement is very similar to a rapid review (to be detailed as the credentialing requirement number 9 listed above) with the exception that the institution is not modifying the actual protocol patient’s treatment, but rather a simulated protocol patient.

## TREATMENT PLANNING PROTOCOL-SPECIFIC BENCHMARK OR DRY RUN CASE(S)

Another approach to verifying that institutions have the ability to comply with protocol requirements is completion of protocol-specific benchmark (aka, dry run) cases. These particular benchmark cases are intended to test the institution’s ability to meet one or more protocol specific requirements such as to contour target or normal tissue structures, create treatment plans that meet prescription requirements, verify that the correct dosimetry parameters are in the treatment planning computer in order to accurately calculate dose, etc. Typically, a protocol-specific benchmark requires download of a CT scan set of a patient considered to be representative of the protocol population. The investigator then follows the guidelines of the protocol in delineating the target volume(s) and producing a treatment plan that complies with the criteria of the protocol. The plan is then submitted for review, usually electronically, and reviewed exactly as a protocol patient would be reviewed. Completion of a protocol-specific benchmark is similar to submission and review of a previously treated patient except that all participants plan on the same CT scan set, resulting in more uniform evaluation of submitted plans.

The benchmark cases are excellent QA tools to assess an institution’s ability to define target volumes per protocol since their definition is as critical to successful protocol participation as is treatment planning and dose delivery. Protocols are usually specific about the volumes to be included in the clinical target volume (CTV), which often may differ from institutional policies. By performing the benchmark case these differences can be highlighted and participants educated as to the requirements of the protocol through feedback from the QA center or protocol PI (Urie et al., 2008).

## MODALITY AND/OR SITE-SPECIFIC BENCHMARK CASE(S)

Treatment modalities and techniques are often common to more than one protocol, and several modality and site-specific benchmark cases have been developed as credentialing tools. For many protocols a modality and/or site-specific benchmark test may be adequate for assessing an institution’s ability to plan a patient in compliance with protocol requirements (Hsu et al., 2010).

The more recently developed benchmark cases of this type may have electronic CT imaging datasets with target volumes embedded in the CT slices. Institutions download the CT scan, plan the treatment as prescribed in the benchmark instructions (target and normal tissues), and submit the treatment plan, usually electronically, for evaluation by a protocol PI or a QA center. There is reciprocity amongst the QA centers and hence cooperative groups such that an institution is required to complete a specific benchmark case only once. The current library of benchmark cases used by the QA centers includes an IMRT case, stereotactic radiosurgery (SRS) case, total body irradiation (TBI) case, brachytherapy cases, breast cases, liver case, MRI-CT fusion case, and a proton case.

As we move toward the future with evidence-based quality assurance, new concepts will be considered, evaluated, and implemented by the QA groups. One effort initiated and completed by members of American Association of Physicists in Medicine’s (AAPM) task group 119 involves multi-institutional radiotherapy and delivery data collection and comparison for quality guidance of clinical IMRT commissioning (Ezzell et al., 2009). A set of mock clinical cases simulating head and neck, prostate, spine cancer and a number of other artificially developed cases designed to test the limits of IMRT planning and delivery systems were downloaded and planned with pre-set criteria. These plans were delivered and verified with QA phantoms and measurement devices from the institutions. Planning dosimetric data were collected and statistically analyzed. Measurements were grouped according to the measurement technique, and statistical analysis was reported as well for each of the techniques. Institutions have been using these datasets and reported results as a benchmark against which to evaluate quantitatively their IMRT planning and delivery systems. This approach has the advantage of simultaneous evaluation of multiple aspects of radiation therapy patient planning that can be performed with the institution’s equipment.

One possible vertical tiered approach could involve parallel data collection initially with RPC and the dry run data submission. Adjustment of the QA credentialing process can be made during the study when data collected has reached statistical significance to suggest either continuing with the dual QA process or reducing the processes to only one. Specific protocols can also be adjusted to accept previous credentialing strategies providing the protocol is not asking a new modality question or change in target volume definition.

## PHANTOM IRRADIATION STUDY

Unlike the benchmark cases and the use of a previous patient treated in the manner specified by a protocol, which are meant to assess the treatment planning process as it relates to the protocol, the phantom irradiation study assesses the complete treatment process for a specific treatment modality that might be common to many protocols (e.g., IMRT). This thorough QA test of the treatment process includes imaging, treatment planning, setup for treatment, and actual delivery of the treatment. The goal of radiotherapy QA for clinical trials is to establish consistency in treatment delivery across a large number of participating institutions. The phantoms provide a consistent test to evaluate each institution’s ability to deliver a specific radiotherapy treatment that ensures that all participating institutions pass the same QA criteria (Ibbott et al., 2006; Kry et al., 2012). As with the benchmark cases, reciprocity exists among the QA centers regarding the phantom irradiation study.

Phantoms have been used to assess SRS, IMRT, SBRT, VMAT, moving targets, and proton therapy through the use of anthropomorphic head, pelvis, liver, thorax, and spine phantoms. For a specific anatomical phantom irradiation study: all of the phantoms are built exactly the same, they all use the same dosimeters (TLD and radiochromic film), they are all evaluated using the same established reading system, they are all analyzed using the same analysis software and they are all judged using the same passing criteria. Unlike relying on each institution’s own QA tests which are highly variable among institutions, dosimetry, evaluation, and passing criteria, the phantom irradiation credentialing requirement is a QA audit that is constant from one institution to the next helping to ensure radiotherapy treatment delivery consistency for clinical trials. It provides a level of radiotherapy treatment delivery QA consistency for multi-institutional clinical trials not obtained elsewhere. The specific phantom irradiation test required for credentialing is detailed within each protocol and is requested through the RPC (Ibbott et al., 2008).

## IMAGE GUIDANCE STUDY

Credentialing for the use of image-guided radiation therapy (IGRT) for radiation therapy protocols can verify a number of aspects of this important validation of treatment volume. For example, the credentialing procedure can verify physical agreement or software corrected agreement between the reference coordinate systems for the treatment device and the imaging equipment. Also, the credentialing process can verify the accuracy of patient linear shifts and possible rotational corrections as determined and implemented by the IGRT system. All systems have the ability to register datasets from various imaging modalities, and verification of this aspect is important. Additionally, the verification can include testing of an institution’s use of the software provided for image registration (Middleton et al., 2011).

Although it might be possible to build all of the credentialing aspects mentioned above into a single credentialing procedure, it is not clear that this approach is either desirable or practical. The RTOG QA office started a procedure for IGRT credentialing in 2009. A key feature of the overall program for IGRT credentialing is verification of each institution’s understanding and adherence to instructions in protocols relating to the way image registration is to be applied. Each study identified as an IGRT protocol includes instructions for using IGRT. The instructions provide guidance on considerations like: the extent of the patient’s anatomy to be included in the registration process, the type of registration to be employed (soft tissue, bone, other), action tolerance levels, and the handling of rotational corrections.

## PRE-TREATMENT CLINICAL AND/OR DOSIMETRIC REVIEW OF AN INDIVIDUAL PROTOCOL PATIENT (RAPID REVIEW, REAL-TIME REVIEW, OR TIMELY REVIEW)

One of the most useful credentialing requirements is the protocol patient pre-treatment clinical and/or dosimetric review by a protocol PI or QA center. Various names are commonly used for this type of testing: rapid, real-time, or pre-treatment review. A related process is the timely review or on-treatment review. The difference being that a rapid or real-time review is a review of a protocol patient’s treatment plan before treatment begins while the timely review is a review of the patient’s treatment plan within the first 3 days of treatment. With a timely review, the patient treatment is not delayed but feedback is given to the institution quickly enough to alter the treatment if necessary. In some situations it is not practical to modify the treatment plan. In the case of IMRT treatment plans, the timely review process can be used to communicate information that improves future plans submitted by the same institution. For both review processes discussed in this subsection, the key feature is the communication of treatment planning and contouring information to the institution and more specifically to the treating clinician. QARC, RTOG QA, ITC, and the RPC are involved in facilitating or conducting these rapid reviews (Bekelman et al., 2012). The requirement to submit patient plan data electronically significantly facilitates the rapid review process. The QA centers have developed tools to permit clinicians to review these cases from their home institutions as well as at the QA centers. These rapid, real-time, and timely reviews provide a consistent evaluation of the treatments ensuring comparability across all institutions entering patients onto a specific trial, much like the phantom irradiation study (Dharmarajan et al., 2012). An added benefit for the review techniques discussed here is the ability to identify deficiencies in the protocol that lead to errors in its implementation. The early reviews can lead to amendment of the protocols or the development of a list of frequently asked questions aimed at decreasing protocol deviations (Chen et al., 2012).

Institutions participating in NCI funded clinical trials typically treat their patients with radiation therapy defined by the broad spectrum of reasonable practice strategies. However, for multi-institutional clinical trials, treatments need to be comparable among all participating institutions (Urie et al., 2008). Data from Peters et al. (2010) demonstrated the benefit of protocol compliant radiation therapy treatment. The study indicated that, even if the trial is not asking a specific radiation question, the radiotherapy component is still vital to the trial conduct and can have direct impact on the outcome of the study. The benefit of the additional effort expended by institutions in the credentialing process helps to ensure that the clinical trial radiotherapy treatments are accurate and comparable. These QA efforts result in trial outcomes that have not been obscured by uncertainty in the radiotherapy treatment data.

## FUTURE

All of the NCI funded cooperative clinical trial study groups and QA centers believe that credentialing has significantly increased the compliance with protocol requirements. This, in turn, serves to validate clinical trial outcome. Credentialing is highly cost efficient as it is far more expensive with significant time lost if clinical trial data is invalidated by poor quality and non-uniform patient treatment. Poor credentialing and non-uniform treatment can invalidate clinical trial data. Lack of guidelines in clinical trial charter can lead to extraordinary disparities in clinical trial conduct (FitzGerald et al., 2008; FitzGerald, 2012; Peters et al., 2010). The quality of the clinical trial studies has improved significantly and the number of deviations has decreased assuring a uniformly treated study population. Hence credentialing will continue, but what and how it is performed will continue to evolve. When new technologies or treatment strategies are introduced into clinical trials, non-uniform application of the technology is routinely seen on quality assurance analysis during the initial phase of the trial. Coupled with staff changes and new equipment purchases, credentialing will remain a self-renewal process insuring both acceptance of institutions into clinical trials and maintenance of practitioner and institution skill set. One aspect of this evolution is the education process that helps institutions better understand what is required relative to treatment planning with special attention to the segmentation of critical structures and targets. The development of site-specific atlases is one example of the teaching tools that can be used as a powerful educational tool. This will not replace credentialing, however will serve to support conduct of the trial and limit deviations on study.

Radiotherapy technology and treatment execution is likely to become even more complex and computer-driven. As these new technologies are introduced into the radiation therapy community, verification of their appropriate use for protocol patients will continue to be necessary. Accomplishing the objective will require rethinking of current approaches.

Essential to credentialing efforts will be appropriateness of the requirements and their validity for multiple groups and protocols. Credentialing must not be overly burdensome to the participants and must be an efficient process for the QA centers while still assuring NCI and the cooperative clinical trial groups that participating radiotherapy facilities deliver comparable radiation doses per protocol specifications.

Dosimetric credentialing for a particular modality or technology will be an effort by the institution that will validate it for all cooperative groups. Similar to the phantom studies currently required by many protocols, successfully completing a credentialing process will allow participation in multiple studies. A universal database, accessible to the institutions as well as to the QA centers, will be used to track and record all credentialing statuses. Issues to be addressed include (1) what triggers a requirement for credentialing and (2) should there be sunset clauses that would at least require the QA centers to evaluate the appropriateness of the exercise after a defined period of time.

Target volume and normal tissue credentialing may need to be disease site-specific. Current approaches to this issue include distribution of a protocol appropriate CT dataset, review of an institution’s treatment of a prior patient who would have been protocol appropriate, and pre-treatment review of the actual protocol patient. The first approach provides a consistent dataset for evaluation and education, while the other two provide data on specific institutions’ patients. A consensus among the QA centers will need to be achieved so that at the very least reciprocity among the groups and protocols for similar disease sites can be achieved. Atlases and web-based educational tools will aid in this process.

A tiered approach to credentialing for specific protocols may be implemented. Protocols with quality of life endpoints may require less rigorous credentialing relative to protocols testing advanced technology therapy. Protocols may be possibly divided into four categories based on the specific protocol question and the radiotherapy modalities permitted: (1) radiotherapy as adjuvant care, (2) standard (which will need to be defined) external beam radiotherapy delivery, (3) external beam radiotherapy and brachytherapy or brachytherapy alone, and (4) new radiotherapy delivery modality or radiation dose calculation technique. Depending on the tier, the rigor and format of the credentialing may be adjusted.

The dosimetric aspects of credentialing are primarily institutional; the target volume aspects of credentialing are primarily individual (clinicians). These considerations raise several questions. Should credentialing be separated into the two aspects? Should clinicians be credentialed individually? Would the credentialing move with them if they move to another institution? How the QA centers handle the separation of the dosimetric and clinical aspects will need to be resolved.

Imaging is a critical component of many modern protocols. Various diagnostic studies, including CT, MRI, and PET, taken at various times in the patient’s overall treatment may be essential for protocol eligibility, target volume definition, and protocol strata as radiotherapy clinical trials embrace the concept of adaptive radiation therapy (ART). The quality of these studies must be adequate to answer the protocol questions with minimal uncertainty. QA centers will need to develop credentialing techniques for imaging quality commensurate with imaging objectives of IROC. It is anticipated credentialing may be performed by an imaging group and that it would apply to all protocols and groups similar to that proposed for a radiotherapy group. Often the images used to generate the targets for radiation therapy treatment volumes can be used to target annotation for patient response on the same study. Integrated imaging and radiation therapy treatment informatics platforms can facilitate these interactions moving forward.

International participation in clinical trials is a high priority of the NCI. But credentialing for participation creates a series of challenges since radiation therapy practice varies substantially around the world. Regulations vary among countries and conventions and procedures vary among countries and non-US clinical trial groups. Even something seemingly simple, such as the required frequency of an external radiation dosimetric audit, may cause significant delay in international participation. The new QA center(s) will need to be mindful of these variations and work with other global QA offices in an attempt to harmonize QA efforts for these international trials while maintaining consistency in clinical trial patient treatments. The quality assurance centers have considerable experience in international collaboration in both group and industry sponsored clinical trials, therefore building upon this experience will be important moving forward as international participation becomes imbedded in the daily work scope of the quality assurance centers (Trimble et al., 2009).

Going forward with the new clinical trial study group and QA center structure, credentialing must be efficient and effective. The goal remains to minimize deviations so that trials are completed with the most consistent and accurate data with the maximum number of evaluable patients in order to validate clinical trial outcome. Credentialing helps to ensure that institutions participating in multi-institutional cooperative clinical trials are meeting protocol specifications in a comparable manner such that deviations are kept low and the maximum amount of patient data is available for analysis.

## Conflict of Interest Statement

The authors declare that the research was conducted in the absence of any commercial or financial relationships that could be construed as a potential conflict of interest.
